# Zfra affects TNF-mediated cell death by interacting with death domain protein TRADD and negatively regulates the activation of NF-κB, JNK1, p53 and WOX1 during stress response

**DOI:** 10.1186/1471-2199-8-50

**Published:** 2007-06-13

**Authors:** Qunying Hong, Li-Jin Hsu, Lori Schultz, Nicole Pratt, Jeffrey Mattison, Nan-Shan Chang

**Affiliations:** 1 Institute of Molecular Medicine, National Cheng Kung University Medical College, Tainan, Taiwan, ROC; 2 Department of Microbiology and Immunology, National Cheng Kung University Medical College, Tainan, Taiwan, ROC; 3 Center for Gene Regulation and Signal Transduction Research, National Cheng Kung University Medical College, Tainan, Taiwan 70101, ROC; 4 Guthrie Research Institute, Laboratory of Molecular Immunology, 1 Guthrie Square, Sayre, PA 18840, USA

## Abstract

**Background:**

Zfra is a 31-amino-acid zinc finger-like protein, which is known to regulate cell death by tumor necrosis factor (TNF) and overexpressed TNF receptor- or Fas-associated death domain proteins (TRADD and FADD). In addition, Zfra undergoes self-association and interacts with c-Jun *N*-terminal kinase 1 (JNK1) in response to stress stimuli. To further delineate the functional properties of Zfra, here we investigated Zfra regulation of the activation of p53, WOX1 (WWOX or FOR), NF-κB, and JNK1 under apoptotic stress.

**Results:**

Transiently overexpressed Zfra caused growth suppression and apoptotic death of many but not all types of cells. Zfra either enhanced or blocked cell death caused by TRADD, FADD, or receptor-interacting protein (RIP) in a dose-related manner. This modulation is related with Zfra binding with TRADD, NF-κB, JNK1 and WOX1, as determined by GST pull-down analysis, co-immunoprecipitation, and mapping by yeast two-hybrid analysis. Functionally, transiently overexpressed Zfra sequestered NF-κB (p65), WOX1, p53 and phospho-ERK (extracellular signal-activated kinase) in the cytoplasm, and TNF or UV light could not effectively induce nuclear translocation of these proteins. Zfra counteracted the apoptotic functions of Tyr33-phosphorylated WOX1 and Ser46-phosphorylated p53. Alteration of Ser8 to Gly abolished the apoptotic function of Zfra and its regulation of WOX1 and p53.

**Conclusion:**

In response to TNF, Zfra is upregulated and modulates TNF-mediated cell death via interacting with TRADD, FADD and RIP (death-inducing signaling complex) at the receptor level, and downstream effectors NF-κB, p53, WOX1, and JNK1.

## Background

Human *WWOX/FRA16D *gene encodes a candidate tumor suppressor WW domain-containing oxidoreductase, designated WWOX, FOR, or WOX1 [1-3]. This gene is located on a common fragile site ch16q23.3–24.1 [1,2]. Loss of heterozygosity (LOH) of *WWOX *gene has been found in several types of cancers [[4,5]; reviews]. WWOX/FOR/WOX1 possesses two *N*-terminal WW domains (containing conserved tryptophan residues), a nuclear localization sequence (NLS) between the WW domains, and a *C*-terminal short chain alcohol dehydrogenase/reductase (SDR) domain. *WWOX *mRNA may undergo alternative splicing, thereby generating at least 8 mRNAs mainly coding for proteins with altered SDR domain sequences [4]. Several protein isoforms have been identified [4]. Nonetheless, presence of specific protein isoforms in normal and cancerous tissues remains to be established.

WWOX/FOR/WOX1 is considered as a candidate tumor suppressor and proapoptotic protein, whereas its *in vivo *function is largely unknown [4]. Under stress conditions, WOX1 undergoes phosphorylation at Tyr33 and may translocate to the mitochondria and nuclei to induce apoptosis in cultured cells and in rat eyes [3,6-9]. Tyr33-phosphorylated or activated WOX1 binds to the proline-rich region and phospho-Ser46 of p53, and both proteins induce apoptosis synergistically [3,6,8]. When WOX1 is functionally suppressed by antisense mRNA, small interfering RNA (siRNA), or dominant negatives, the stability of p53 and its apoptotic function are significantly suppressed [3,6,8]. The proapoptotic function of WOX1 is probably associated, in part, with its interaction with p53, p73, JNK1 and other transcription factors [4].

To isolate WOX1-binding proteins using yeast two-hybrid cDNA library screening [3,6,8], we found that WOX1 interacts with a small size 31-amino-acid protein, Zfra (zinc finger-like peptide that regulates apoptosis) [10]. Zfra belongs to the family of C2H2 type zinc finger proteins [11-13], and has a sequence homology to transcription factor forkhead protein xFKHR1 [14]. Zinc finger proteins interact with DNA and RNA, which is essential for regulating gene transcription during cell growth and embryogenesis. Damage to the zinc fingers in DNA repair proteins may induce carcinogenesis [15]. Zfra mRNA is expressed in many organs and tissues and most abundant in the spleen [10]. However, it is absent in several prostate and breast cancer cell lines [10].

Zfra participates in the signal pathway of tumor necrosis factor (TNF or TNF-α) [[16,17]; reviews]. Zfra appears to play a dual role in regulating the cytotoxic effects of TNF and Fas ligand (FasL) [10]. Zfra either enhances or blocks the apoptotic functions of transiently overexpressed receptor adaptor proteins TRADD (tumor necrosis factor receptor type 1-associated death domain protein) and FADD (Fas-associated death domain-containing protein) [10]. TRADD and FADD are recruited to the TNF receptors when cells are stimulated with TNF or Fas ligand (FasL). In response to TNF and UV light, Zfra undergoes self-binding and interacts with JNK1 [10]. JNK1 is a downstream effector of the TNF signaling [18-20]. The functional mechanism for the action of Zfra remains to be established.

In this study, we further investigated the underlying mechanisms for the regulatory effect of Zfra on cell death caused by transiently overexpressed death domain proteins, including TRADD, FADD and RIP (receptor-interacting protein). We determined the role of a conserved phosphorylation site at serine 8 in conferring Zfra-induced apoptosis. Also, we examined whether Zfra regulates the activation of transcription factor NF-κB and tumor suppressors p53 and WOX1 in response to TNF and UV light, and discussed the biological implications of their interactions both *in vitro *and *in vivo*.

## Results

### Transiently overexpressed Zfra induces apoptosis

We have previously shown that when ectopic Zfra is stably expressed in L929 fibroblasts, these cells resist the cytotoxic effects of TNF and FasL [10]. In contrast, depending upon the concentrations used or the extent of expression, transiently expressed Zfra could either enhance or inhibit the cytotoxic function of overexpressed death domain proteins TRADD and FADD [10]. The underlying mechanism of this regard is unknown.

**Figure 1:**
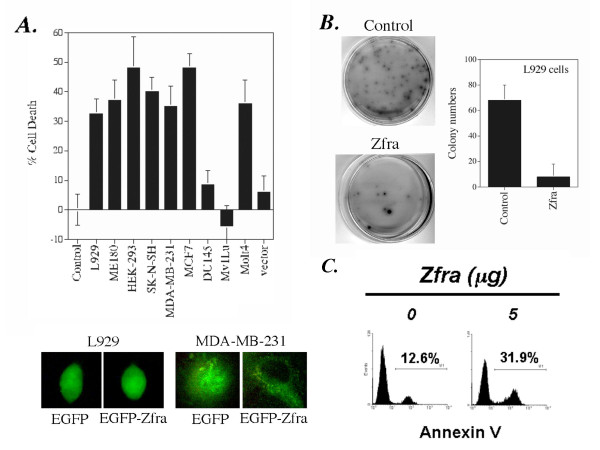
**Zfra induces apoptotic cell death**. (**A**) Transiently overexpressed Zfra induced death of human ovarian ME180, embryonic kidney HEK-293, neuroblastoma SK-N-SH, breast MDA-MB-231 and MCF7 cells, Molt4 T lymphocytes and murine L929 fibroblasts, but not human prostate DU145 and mink lung epithelial Mv1Lu cells. These cells were transfected with an EGFP (vector) or EGFP-Zfra expression vector (150 ng/well in 96-well microtiter plates) or medium (control) by CaPO4, followed by culturing for 48 hr and then staining with crystal violet for determining the extent of death (n = 8; mean ± standard deviation; *p *< 0.001, EGFP vector-transfected cells versus EGFP-Zfra-transfected cells except DU145 and Mv1Lu; Student's t tests). Molt4 T cells were transfected with the DNA constructs by electroporation. Data shown in the buffer and vector (EGFP) controls are from testing L929 cells. Similar data were obtained using other cells. The numbers of cells with positive EGFP or EGFP-Zfra protein expression were ~60–75%, as determined by fluorescence microscopy. EGFP-Zfra was present ubiquitously in cell compartments in majority of the tested cells (see representative L929 cells; 200× magnification). Interestingly, EGFP-Zfra was mainly present in the cytoplasm of MDA-MB-231 cells (400× magnification). (**B**) In adherence- or anchorage-independent growth assay on agarose, Zfra was shown to block colony formation of L929 cells, as opposed to empty vector controls (n = 3, *p *< 0.001; Student's t test). Live colonies were stained with a soluble tetrazolium-based MTS proliferation reagent. (**C**) Molt4 T cells were electroporated with the EGFP-Zfra expression vector, followed by culturing for 48 hr and analyzing the extent of apoptosis using Annexin V assay.

To further examine the role of Zfra-mediated death, a panel of adherent cell lines was transfected with an EGFP-tagged Zfra cDNA expression construct by CaPO4. For non-adherent Molt4 T cells, the DNA construct was introduced by electroporation (Figure 1A). In controls, cells were transfected with buffer or EGFP vector only. Following 48 hr in culture, Zfra induced death of human cell lines including ovarian ME180, embryonic kidney HEK-293, neuroblastoma SK-N-SH, Molt4 T lymphocytes, and breast MCF7 and MDA-MB-231 cells, and murine L929 fibroblasts (Figure 1A). However, Zfra had no effect on human prostate DU145 and mink lung epithelial Mv1Lu cells (Figure 1A). Based on our previous observations [10], an optimal dose of Zfra, 150 ng/well in 96-well plates, was used to produce ~30–40% death of L929 cells. Cells with positive protein expression were approximately 60–75%, as determined by fluorescence microscopy (Figure 1A). EGFP-Zfra was present ubiquitously in cell compartments in majority of the tested cells. Interestingly, EGFP-Zfra was mainly present in the cytoplasm in breast MDA-MB-231 cells (Figure 1A).

Cells, including HEK-293, SK-N-SH, Molt4 and L929, express Zfra mRNA, and they are sensitive to Zfra-induced death [10]. Breast MCF7 and MDA-MB-231 cells do not express Zfra [10], but were sensitive to Zfra-mediated death (Figure 1A). Prostate DU145 is a Zfra-negative cell line [10], and resisted death by Zfra (Figure 1A). Together, our data show that resistance to Zfra-mediated death is not related with the presence or absence of endogenous Zfra in cells.

We examined the effect of Zfra on anchorage-independent cell growth [21]. As expected, Zfra blocked colony formation of L929 cells on agarose plates, as compared to empty vector controls (Figure 1B).

To further determine Zfra-mediated apoptosis, Molt4 T cells were transfected with EGFP-Zfra and cultured for 48 hr. The extent of exposure of phosphatidylserine to cell surface was determined by Annexin V assay. Zfra increased the exposure of phosphatidylserine on cell surface (Figure 1C), indicating that cells have undergone apoptosis.

### Involvement of conserved serine 8 (Ser8) in Zfra-induced apoptosis

**Figure 2:**
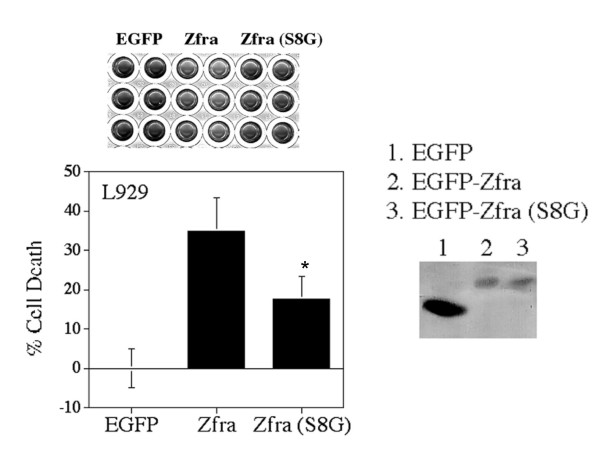
**Ser8 is involved in Zfra-induced apoptosis**. Ser8 is a conserved phosphorylation site in Zfra [10]. Ser8 was altered to Gly (S8G). L929 cells were electroporated with EGFP-Zfra, S8G, or EGFP, followed by culturing for 48 hr and staining with crystal violet. Transiently overexpressed Zfra induced death of L929 cells (34.8 ± 8.6%; n = 8), whereas S8G mutant had a significantly reduced activity in causing cell death (17.1 ± 5.6%; n = 8; *p *< 0.005, Student's t test; see *). The extent of protein expression is shown.

Ser8 is a conserved phosphorylation site in Zfra [10]. We determined the potential role of this site in conferring the apoptotic function of Zfra. By site-directed mutagenesis, Ser8 was altered to Gly (S8G). L929 cells were electroporated with EGFP-Zfra, S8G, or EGFP, and the cells were cultured for 48 hr. Again, transiently overexpressed Zfra induced death of L929 cells (34.8 ± 8.6%; n = 8), whereas S8G mutant had a significantly reduced activity in causing cell death (17.1 ± 5.6%; n = 8) (Figure 2). These observations suggest that Ser8 phosphorylation is essential for Zfra-induced apoptosis.

### Zfra regulates death domain protein-mediated cell death

**Figure 3:**
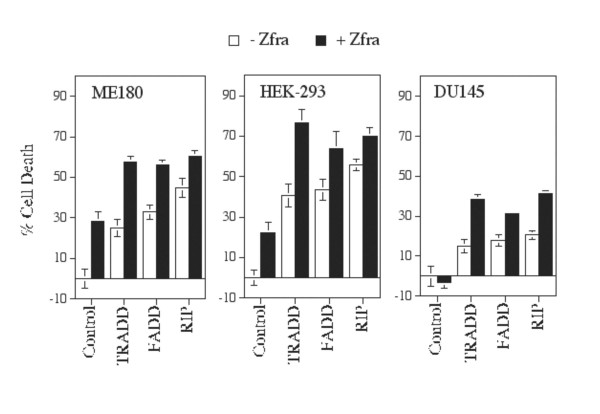
**Effect of Zfra on TRADD, FADD, or RIP-mediated cell death**. ME180, HEK-293 and DU145 cells were transfected with cytotoxic doses of TRADD, FADD, or RIP cDNA (200 ng/well), in the presence or absence of EGFP-Zfra (200 ng/well) by CaPO4. These cells were cultured for 48 hr and then stained with crystal violet. In Zfra-sensitive ME180 and HEK-293 cells, Zfra and TRADD increased cell death in an additive manner (n = 8; *p *< 0.001, TRADD alone versus TRADD/Zfra in combination; Student's t tests), whereas no additive effect was shown for Zfra with FADD or RIP (n = 8; *p *> 0.05, FADD or RIP alone versus Zfra/FADD or Zfra/RIP; Student's t tests). In Zfra-resistant DU145 cells, Zfra alone did not induce death, but enhanced the death by ectopic TRADD, FADD and RIP by ~100–150% increases (n = 8; *p *< 0.001; Student's t tests). EGFP-Zfra was expressed in approximately 60–70% of total cells, as visualized by fluorescence microscopy.

TRADD, FADD and RIP are recruited to the TNF receptor during TNF signaling. RIP interacts with TRADD or FADD [17]. We have shown that Zfra modulates the apoptotic function of overexpressed TRADD and FADD [10]. Here, we continued to examine whether Zfra affects RIP-mediated cell death. We used ME180, HEK-293 and DU145 cells, and DU145 was resistant to Zfra-mediated death (Figure 1). These cells were cotransfected with "cytotoxic" doses of cDNA expression constructs of TRADD and/or Zfra (tagged with EGFP) by CaPO4. Similar experiments were performed with Zfra with FADD and RIP. Both Zfra and TRADD induced death of ME180 and HEK-293 cells in an additive manner (Figure 3). Nonetheless, Zfra did not increase cell death with FADD or RIP in an additive manner (Figure 3). In the Zfra-resistant DU145 cells, Zfra alone did not cause cell death, but increased the cytotoxic function of TRADD, FADD and RIP by approximately 100–150% (Figure 3).

**Figure 4:**
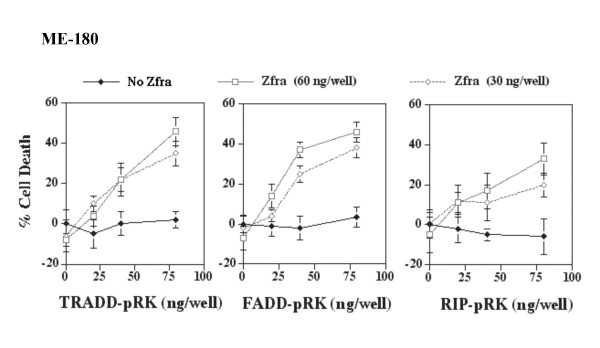
**Synergistic enhancement of cell death by non-cytotoxic levels of Zfra and TRADD, FADD or RIP**. Zfra-sensitive ovarian ME-180 cells were transfected with non-cytotoxic doses of TRADD (FADD or RIP) (20, 40, 80 ng/well) and/or EGFP-Zfra by CaPO4. Zfra and TRADD, FADD, or RIP synergistically induced ME180 cell death in 48 hr (n = 16; mean ± standard deviation; *p *< 0.001; Zfra, TRADD, FADD, or RIP alone versus Zfra/TRADD, Zfra/FADD, or Zfra/RIP; Student's t tests). Cells were stained with crystal violet. EGFP-Zfra was expressed in approximately 60–70% of total cells, as visualized by fluorescence microscopy.

Next, we tested the effect on death using non-cytotoxic doses of cDNA constructs. Under this condition, there was a synergistic enhancement of ME180 cell death by Zfra and TRADD, FADD or RIP (Figure 4).

**Figure 5:**
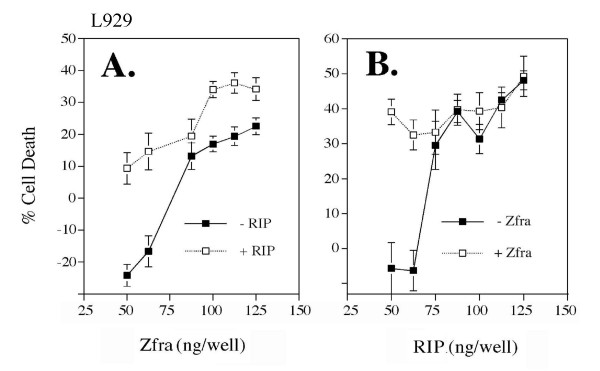
**Zfra regulates cell growth in a biphasic manner**. (**A**) L929 cells were transfected with various amounts of EGFP-Zfra and/or a non-toxic dose of RIP (100 ng/well) by CaPO4. Zfra alone enhanced cell growth at low doses, but induced death at higher doses (n = 8; mean ± standard deviation). RIP blocked the Zfra-mediated cell growth. (**B**) Similarly, L929 cells were transfected with various amounts of RIP and/or a non-toxic dose of Zfra (60 ng/well). At low levels, RIP had little or no effect on cell growth. When in combination, both Zfra and RIP effectively increased the cell death (n = 8; mean ± standard deviation). Cells were stained with crystal violet. EGFP-Zfra was expressed in approximately 60–70% of total cells, as visualized by fluorescence microscopy.

Indeed, Zfra regulates cell growth in a biphasic manner. At low doses, Zfra enhanced growth of Zfra-sensitive L929 cells. But, cell death occurred when higher doses of Zfra were used, as measured 48 hr post transfection (Figure 5A). Cytotoxic levels of RIP blocked the growth enhancement of Zfra. Similarly, at low levels RIP had no effect on L929 cell growth, and when in combination, non-toxic doses of Zfra and RIP increased the cell death (Figure 5B).

Taken together from the above experiments, our data clearly show that depending upon concentrations or the extent of expression, Zfra may regulate the function of death domain proteins TRADD, FADD or RIP in a synergistic or an antagonistic manner.

### Zfra physically interacts with TRADD, and binds to the N-terminal first WW domain and C-terminal SDR domain of WOX1

**Figure 6:**
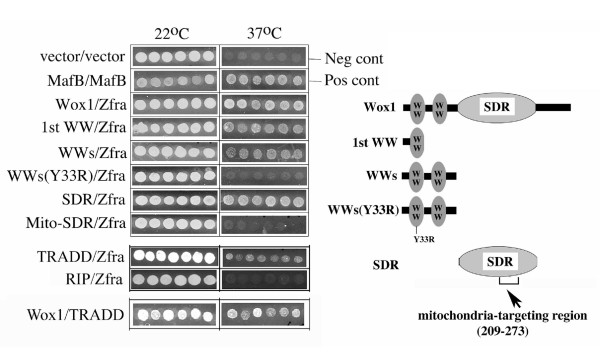
**Zfra physically binds TRADD and the *N*-terminal first WW domain and *C*-terminal SDR domain in WOX1**. Binding of Zfra (as target) with WOX1 (as bait) was mapped by Ras rescue-based yeast two-hybrid analysis (see Materials and Methods). Positive binding is evidenced by the growth of mutant yeast at 37°C in a selective medium. Zfra bound to the *N*-terminal first WW domain and the *C*-terminal SRD domain of WOX1. Alteration of the known phosphorylation site Tyr33 to Arg33 (Y33R) in WOX1 abrogated the binding. Zfra did not interact with the mitochondria-targeting region in the SDR domain (Mito-SDR). No yeast cell growth at 37°C was shown when empty vector versus empty vector was tested as a negative control. In a positive control, the self-binding of MafB is shown. In addition, Zfra was shown to bind the full-length TRADD, but not RIP. WOX1 also bound TRADD. Neg cont: negative control; Pos cont: positive control.

We have utilized a novel cytoplasm-based two-hybrid analysis in mapping protein/protein interactions between domains and/or motifs [3,6,8]. Briefly, bait is expressed in the cytoplasm of yeast cells. When the bait physically interacts with a cell membrane-anchored target, mutant yeast cells grow in a selective medium at 37°C as a result of activation of Ras signal pathway [3,6,8]. We showed that Zfra interacted with WOX1 from yeast two-hybrid library screening. To map the Zfra-binding domain(s) in WOX1, we determined that Zfra physically interacted with the *N*-terminal first WW domain of WOX1 (Figure 6). Alteration of the known phosphorylation site Tyr33 to Arg in this domain abrogated the binding (Figure 6), suggesting that Tyr33 phosphorylation is needed for WOX1 to interact with Zfra. During activation, WOX1 undergoes Tyr33 phosphorylation and nuclear translocation both *in vitro *and *in vivo *[3,6,8,9]. Zfra also bound the *C*-terminal SDR domain. This domain has a mitochondria-targeting region, and Zfra did not bind to this region (Figure 6). In a negative control, empty vector versus empty vector was tested. In a positive control, self-binding of MafB is shown [3,6,8] (Figure 6).

We also determined that Zfra physically interacted with the full-length TRADD but not RIP, and WOX1 interacted with TRADD (Figure 6). Together, these observations suggest that Zfra and WOX1 may complex with TRADD, and this complex affects TNF-mediated cell death.

### TNF induces the binding of Zfra with Tyr33-phosphorylated WOX1, JNK1, and NF-κB

**Figure 7:**
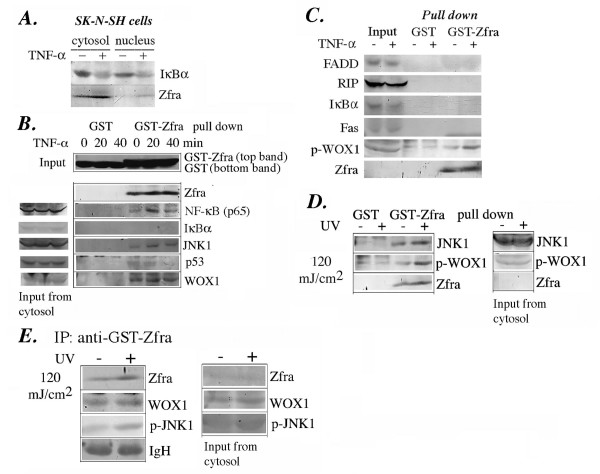
**TNF and UV light increase the binding of Zfra with WOX1, JNK1 and NF-κB, but weakly with p53 in SK-N-SH cells**. (**A**) Exposure of SK-N-SH cells to TNF (50 ng/ml) for 40 min resulted in upregulation of Zfra and degradation of IκBα. (**B**) In GST pull-down analysis using SK-N-SH cells, TNF increased the binding of GST-Zfra with NF-κB (p65) and JNK1 (greater than 50%), and weakly with p53 but not IκBα. GST-Zfra physically interacted with endogenous Zfra and WOX1, and TNF limitedly increased the binding (less than 30%). In negative controls, GST alone could not bind the above-indicated proteins. The relative amounts of GST and GST-Zfra used in the pull down are shown. One-twentieth amounts of protein input for endogenous Zfra and other indicated proteins are shown in Western blotting. (**C**) Similarly, during a 10-min treatment, TNF increased the binding of GST-Zfra with p-WOX1, but not with FADD, RIP, IκBα and Fas. (**D**) SK-N-SH cells were exposed to UV light (120 mJoule/cm^2^) and then cultured for 1 hr, followed by processing GST pull-down analysis. UV light increased the binding of endogenous Zfra with p-WOX1 and JNK1. (**E**) By co-immunoprecipitation, UV light (120 mJoule/cm^2^) increased the binding of endogenous Zfra with itself and p-JNK1 (at Thr183/Tyr185) (greater than 50% increase), but limitedly with WOX1 (less than 20% increase), in SK-N-SH cells.

To verify the above observations, we utilized GST (Glutathione S-Transferase) pull-down analysis and co-immunoprecipitation to characterize the binding of Zfra with its partners. Zfra was tagged with GST at its *N*-terminus. Recombinant GST-Zfra protein was produced in bacteria, and then purified using glutathione-Sepharose 4B resin. Similarly, recombinant GST alone was produced and purified. Exposure of Zfra-sensitive SK-N-SH cells to TNF resulted in an increased expression of Zfra and degradation of IκBα (Figure 7A), indicating that this cell line has a functional TNF signal pathway. This cell line expresses a low level of Zfra.

Zfra is able to undergo self-association and binds JNK1 [10]. In GST pull-down assays, GST-Zfra was shown to interact with endogenous Zfra and WOX1, and TNF barely increased the binding in SK-N-SH cells (Figure 7B). In addition, TNF increased the binding of GST-Zfra with JNK1, NF-κB (p65) and p-WOX1 (Tyr33 phosphorylated), and weakly with p53 in a time-related manner (Figure 7B and 7C). Zfra could not interact with IκBα, FADD, RIP and Fas (Figure 7B and 7C). Similarly, in response to UV light (120 mJoule/cm^2^), there was an increased binding of GST-Zfra with endogenous Zfra, p-WOX1 and JNK1 in SK-N-SH cells, as determined by pull-down analysis (Figure 7D). UV light increased the binding of endogenous Zfra with phosphorylated JNK1 (at Thr183/Tyr185) in SK-N-SH cells, as determined by co-immunoprecipitation (Figure 7E).

### Transiently overexpressed Zfra sequesters WOX1, NF-κB, p53 and p-ERK in the cytoplasm

**Figure 8:**
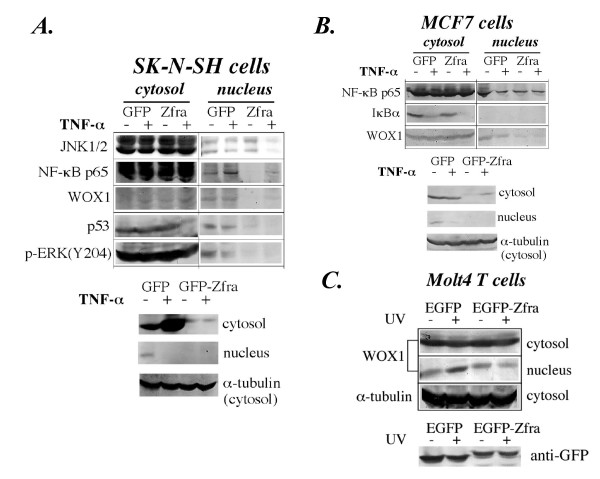
**Transiently overexpressed Zfra sequesters NF-κB, WOX1, p53 and ERK in the cytoplasm**. Breast MCF7 and neuroblastoma SK-N-SH cells were electroporated with an EGFP-Zfra construct or EGFP only, cultured overnight, and exposed to TNF for 40 min. Expression of GFP and GFP-Zfra is shown (**A, B**). (**A**) Without stimulation, EGFP-Zfra alone inhibited nuclear localization of NF-κB (p65), WOX1, p53 and p-ERK (Y204 phosphorylated) in SK-N-SH cells, whereas it had little or no effect on JNK1/2. TNF (50 ng/ml) could not induce nuclear translocation of endogenous JNK1/2, NF-κB (p65), p-ERK, p53 and WOX1 in Zfra-expressing cells during treatment for 40 min. (**B**) Similarly, in MCF7 cells, EGFP-Zfra suppressed nuclear localization of NF-κB and WOX1, and TNF could not induce nuclear translocation of these proteins. (**C**) EGFP-Zfra did not have a significant effect on the nuclear localization of WOX1 in Molt4 T cells; however, it blocked UV light-induced nuclear translocation of WOX1. Ectopic expression of EGFP and EGFP-Zfra in the above cells is shown in each panel. The levels of cytosolic α-tubulin are regarded as loading controls.

The above data show that endogenous Zfra may physically interacts with endogenous WOX1, NF-κB and JNK1/2 in non-stimulated cells. We examined whether transiently overexpressed Zfra sequesters these proteins in the cytoplasm and prevents their nuclear translocation by stress stimuli. SK-N-SH cells were electroporated with EGFP-Zfra or EGFP only, followed by culturing for 24 hr. Transiently overexpressed EGFP-Zfra suppressed nuclear localization of NF-κB (p65), WOX1, p53, p-ERK (extracellular signal-regulated kinase), but had little or no effect on JNK1/2 (Figure 8A). TNF induced nuclear translocation of NF-κB in EGFP control cells, but this event was blocked in the EGFP-Zfra-expressing cells (Figure 8A). Similarly, in Zfra-negative breast MCF7 cells, transiently overexpressed EGFP-Zfra suppressed nuclear translocation of NF-κB and WOX1 (Figure 8B). Interestingly, transiently overexpressed EGFP-Zfra did not have a significant effect on the nuclear localization of WOX1 in Molt4 T cells (Figure 8C). However, EGFP-Zfra blocked UV light-induced WOX1 nuclear translocation in Molt4 T cells.

### Zfra counteracts the apoptotic function of WOX1

**Figure 9:**
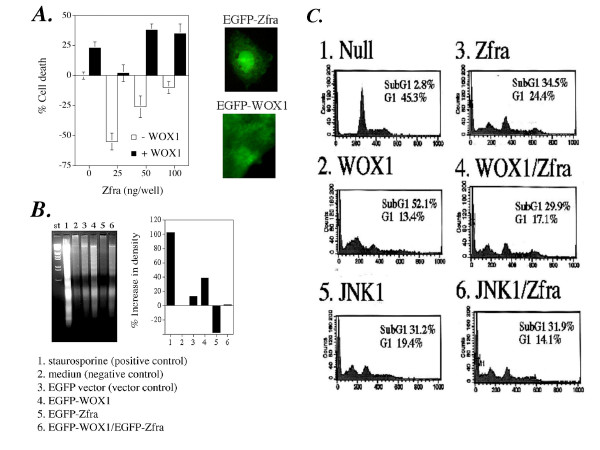
**Zfra antagonizes the apoptotic function of WOX1 and JNK1**. **(A) **L929 cells were transfected with a cytotoxic dose of EGFP-WOX1 in the presence or absence of low doses of EGFP-Zfra by CaPO4 (n = 8). The cells were cultured 24 and 48 hrs and stained with crystal violet. Zfra blocked WOX1-mediated growth suppression and death in 24 hr (data not shown for 48 hr). Expression of EGFP-tagged Zfra and WOX1 is shown, as determined by fluorescence microscopy (400× magnification). **(B) **Similarly, L929 cells were electroporated with WOX1 and/or Zfra, cultured 48 hr, and then processed for DNA fragmentation. An apoptosis-inducing amount of WOX1 and a non-apoptosis-inducing dose of Zfra were used. In a positive control, cells were treated with staurosporine (stauro; 500 nM) for 16 hr. In negative controls, cells were electroporated with medium or GFP vector only. ep, electroporation. **(C) **L929 cells were electroporated with an apoptosis-inducing amount of WOX1 and/or Zfra, or JNK1 (or medium only), and cultured for 24 and 48 hrs, respectively. Apoptosis occurred in 24 hr, as evidenced by increased percentages of cells in the subG1 phase from cell cycle analysis (data not shown for 48 hr). WOX1, JNK1, or Zfra alone suppressed cell growth, as indicated by reduced cell populations at the G1. However, both Zfra and WOX1 or JNK1 could not increase apoptosis in a synergistic manner. X-axis: DNA content; Y-axis: Cell numbers.

Next, we examined whether Zfra counteracts the apoptotic function of WOX1. L929 cells were transfected with an apoptosis-inducing amount of EGFP-WOX1, in the presence or absence of low doses of EGFP-Zfra by CaPO4. In parallel with the above observations (Figure 4), low doses of Zfra enhanced the growth of L929 cells during 24 and 48 hr in culture (Figure 9A; data not shown for 48 hr). Zfra counteracted WOX1-mediated growth suppression and death of L929 cells, as determined using crystal violet staining (Figure 9A). Similar results were observed by counting the number of apoptotic nuclei (stained with DAPI; data not shown). Protein expression of EGFP-WOX1 and EGFP-Zfra were examined by fluorescence microscopy (Figure 9A). WOX1 is shown in a perinuclear area, whereas Zfra is expressed in both cytoplasm and nucleus.

Similarly, L929 cells were electroporated with WOX1 and/or Zfra. These cells were cultured for 24 hr, and then processed for DNA fragmentation in agarose gel electrophoresis. WOX1-induced apoptosis was blocked by a non-apoptotic dose of Zfra (Figure 9B).

Again, L929 cells were then electroporated with apoptosis-inducing amounts of WOX1 and/or Zfra, followed by culturing for 24 and 48 hr, and then processing for cell cycle analysis by FACS. WOX1 or Zfra alone induced growth suppression and apoptosis, as evidenced by increases in the SubG1 phase and decreases in the G1 phase of the cell cycle in cell populations post transfection for 24 hr (Figure 9C; data not shown for 48 hr). However, when in combination, Zfra and WOX1 failed to synergistically increase cell death (Figure 9C). Similar results were observed by testing Zfra with JNK1 (Figure 9C). Overall, Zfra physically interacts with WOX1 and JNK1, and may counteract the apoptotic function of these proteins under stress conditions.

### Phosphorylation at Tyr33 is essential for WOX1 to counteract Zfra-mediated apoptosis

**Figure 10:**
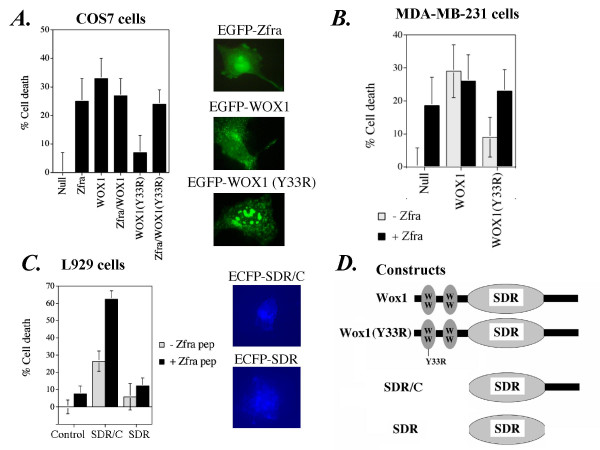
**Phosphorylation of WOX1 at Tyr33 is essential for its apoptotic function and for counteracting Zfra-mediated cell death**. **(A) **Transiently overexpressed EGFP-Zfra and EGFP-WOX1 induced death of COS7 fibroblasts (n = 8, mean ± standard deviation). Zfra counteracted with WOX1 in causing cell death. Alteration of Tyr33 to Arg33 (Y33R) abolished the apoptosis-inducing activity of WOX1, and this mutant did not block Zfra-mediated cell death. Expression of EGFP-tagged Zfra, WOX1 and WOX1(Y33R) is shown in fluorescent micrographs (400× magnification). WOX1 is shown in the perinuclear area, whereas the Y33R mutant tends to aggregate in the nuclei. Zfra is expressed ubiquitously in cells. **(B) **Similar experiments were carried out in breast MDA-MB-231 cells. Again, Zfra and WOX1 did not increase cell death in a synergistic manner (n = 8, mean ± standard deviation). The Y33R mutant lost its apoptotic function and had no apparent effect on Zfra-mediated cell death. **(C) **L929 cells were transfected with the SDR domain plus a *C*-terminal tail (SDR/C) or the SDR domain alone (tagged with ECFP at the *N*-terminus). These cells were cultured for 24 hr in the presence or absence of a synthetic full-length Zfra peptide (10 μM), and the extent of cell death was determined by crystal violet staining. Zfra enhanced cell death caused by SDR/C (n = 8, mean ± standard deviation). SDR alone could not cause cell death, and Zfra did not increase the cell death. Protein expression of ECFP-SDR/C and ECFP-SDR is shown. (**D**) Constructs made for the above experiments are shown.

Phosphorylation of WOX1 at Tyr33 is essential for its apoptotic function [8]. In parallel with the above observations (Figure 9), overexpressed WOX1 did not synergistically increase cell death with Zfra in COS7 cells (Figure 10A). Also, alteration of Tyr33 to Arg (Y33R) abolished the apoptosis-inducing activity of WOX1 (Figure 10A). This Y33R mutant could not bind Zfra (Figure 6), and did not affect Zfra-mediated cell death (Figure 10A). This relationship was further verified in breast MDA-MB-231 cells (Figure 10B).

We tested the function of SDR domain and a *C*-terminal tail (SDR/C) in WOX1 (Figure 10C). L929 cells were transfected with cDNA constructs for expressing SDR or SDR/C, followed by culturing for 24 hr in the presence or absence of a synthetic full-length Zfra peptide (10 μM). Transiently overexpressed SDR/C exerted cell death. Zfra peptide alone had no effect but enhanced cell death caused by SDR/C (Figure 10C). SDR alone could not cause cell death and Zfra and SDR, in combination, did not increase the cell death (Figure 10C). Together, these observations indicate a critical role of Tyr33 phosphorylation in WOX1 in causing cell death and interacting with Zfra. Also, when Zfra binds to the *C*-terminal SDR/C region, both Zfra and WOX1 may enhance cell death in a synergistic manner.

Protein expression of ECFP-SDR/C and ECFP-SDR is shown. The extent of protein expression was in more than 70% of transfected cells, as determined by fluorescence microscopy. Constructs made for the above experiments are shown (Figure 10D).

### Phosphorylation of p53 at Ser46 is essential for counteracting Zfra-mediated apoptosis

**Figure 11:**
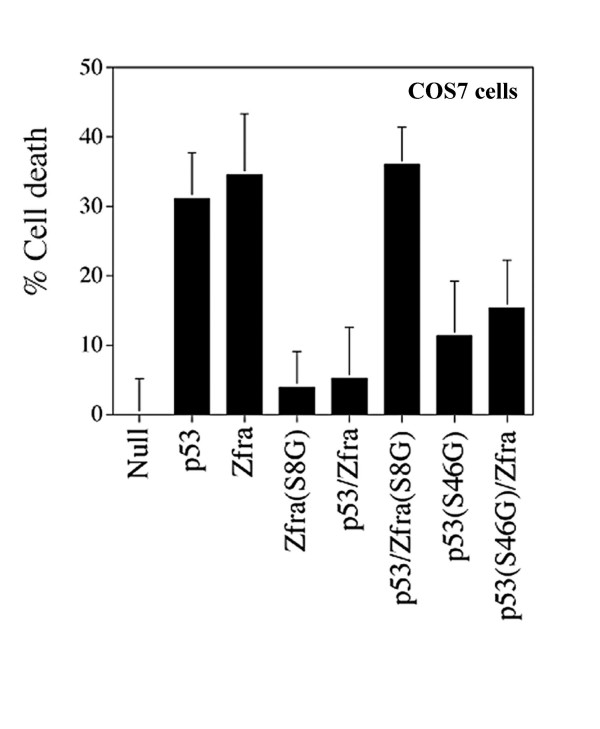
**Phosphorylation of p53 at serine 46 (S46) is essential for counteracting Zfra-mediated cell death**. COS7 cells were co-transfected with both p53 and/or Zfra by electroporation, followed by culturing for 24 hr and then determining the extent of cell death by staining with crystal violet. In addition, the cells were also introduced with mutants of p53 (S46) and/or Zfra(S8G). The results show that p53 and Zfra nullified each other's activity in causing cell death (n = 8, mean ± standard deviation). The apoptotic function of both Zfra(S8G) and p53(S46G) was significantly reduced. S8G mutant had no effect in reducing p53-mediated cell death.

Similarly, overexpressed p53 and Zfra nullified each other's effect in inducing death of COS7 cells (Figure 11). Zfra(S8G) mutant lost its apoptotic function (Figures 2 and 11), and had no effect in counteracting p53-mediated cell death (Figure 11). Mutant p53(S46G) had a reduced activity in causing cell death, and interestingly this mutant also inhibited Zfra's apoptotic function (Fig. 11). The extent of protein expression was more than 70%, as determined by fluorescence microscopy.

## Discussion and conclusion

**Figure 12:**
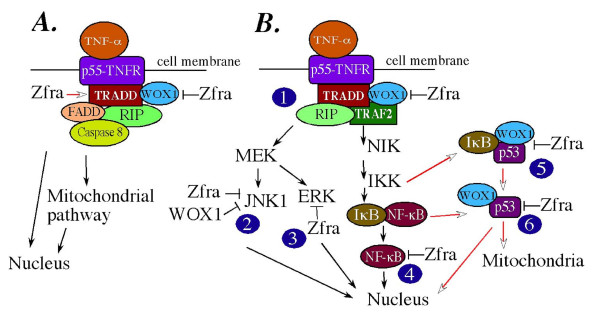
**A schematic model of Zfra/WOX1 involvement in TNF signaling**. TNF is able to initiate two counteractive pathways – one apoptotic and the other protective. **(A) **In the death pathway, binding of TNF to the cognate p55-TNF receptor (TNFR) results in recruitment of death domain proteins TRADD, FADD and RIP, thus generating the so called death-inducing signaling complex (DISC). Caspase 8 and downstream effectors are then activated to induce cell death at the mitochondrial and nuclear levels [16,17]. In this study, we discovered that Zfra physically interacts with TRADD and WOX1, and that WOX1 binds TRADD. Thus, Zfra and WOX1 are likely to be recruited to the DISC. WOX1 enhances TNF cytotoxicity [3], and Zfra counteracts the WOX1 function. Zfra either enhances or inhibits the function of death domain proteins (open arrow). Thus, the ying and yang of cell death depends upon the strength of DISC formation and the counteractive or enhancing force of Zfra. **(B) **In the protective pathway, p55-TNF recruits TRADD, TRAF2 and RIP, followed by activating several downstream adaptors and finally JNK1 and NF-κB. We determined that overexpressed Zfra sequesters p53, WOX1, and NF-κB in the cytoplasm. Thus, Zfra is likely to bind and block the function of these proteins during TNF signaling (see each step marked by a number). In **Step 1**, at the membrane level, Zfra binds TRADD in the presence of TRADD, TRAF2, RIP and WOX1. In **Step 2**, a trimolecular complex of Zfra/JNK1/WOX1 may form when JNK1 is activated by the upstream activated MEK. Zfra binds and counteracts the apoptotic function of JNK1. Also, JNK1 counteracts the apoptotic function of WOX1 [6]. In **Step 3**, MEK activates ERK, and that Zfra may bind and sequester ERK to the cytoplasm. In **Step 4**, phosphorylation of IκBα by IKK causes degradation of IκBα and release of NF-κB for nuclear translocation. Again, Zfra is able to bind and sequester NF-κB in the cytoplasm. In **Step 5 and 6**, TNF induces NF-κB activation, and then NF-κB activates p53 [22]. The non-ankyrin *C*-terminus of IκBα physically interacts with cytosolic p53 [25]. p53 is functionally associated with WOX1, and both proteins may induce apoptosis synergistically [3,4,6,8]. Thus, an *in vivo *complex of Zfra with IκBα/p53/WOX1 or p53/WOX1 is likely (Chang *et al*., submitted).

In this study we have shown that the small size Zfra regulates TNF-mediated cell death via directly binding with TRADD, NF-κB, JNK1, p-ERK and WOX1, and indirectly with FADD, RIP and p53 (Figure 12). Upon binding with p55 TNF receptor (p55-TNFR), TNF engages in two signaling pathways for either protecting cells from death or committing cells to death. For initiating cell death, p55-TNFR recruits TRADD, FADD and RIP to generate a death-inducing signaling complex (DISC) (Figure 12A). Caspase 8 is then recruited to the DISC and becomes activated, followed by activating downstream caspases to induce apoptosis at both the mitochondrial and nuclear levels [16,17].

We determined that Zfra interacts with TRADD but not FADD or RIP (Figure 12A). Zfra affects the cytotoxic function of these death domain proteins via direct and indirect manners (open arrow). Zfra physically interacts with WOX1, and that WOX1 binds TRADD. Thus, Zfra and WOX1 are likely to be recruited to the DISC during TNF signaling (Figure 12A). This assumption has yet to be validated by co-immunoprecipitation and confocal and immunoelectron microscopy.

Previously we have shown that WOX1 enhances TNF cytotoxicity [3]. WOX1 also enhances the cytotoxic function of TRADD [3]. Here we determined that both WOX1 and Zfra counteract with each other in regulating apoptosis. Zfra either enhances or inhibits the function of death domain proteins. Thus, the driving force for committing cells to death in response to TNF is likely coming from a balanced and counter-balanced work among Zfra, WOX1 and DISC.

In the TNF-initiated protective pathway, Zfra is shown to interact with TRADD, JNK1 and NF-κB (Figure 12B). Thus, formation of Zfra-containing regulatory complexes probably occurs in the TNF signaling cascade. In **Step 1**, at the membrane level, Zfra binds TRADD in the presence of TRADD, TRAF2, RIP and WOX1. In **Step 2**, a trimolecular complex of Zfra/JNK1/WOX1 may form when JNK1 becomes activated by the upstream activated MEK. Zfra binds and counteracts the apoptotic function of JNK1. Also, JNK1 counteracts the apoptotic function of WOX1 [6]. In **Step 3**, MEK activates ERK, and that Zfra may bind and sequester ERK to the cytoplasm. In **Step 4**, phosphorylation of IκBα by IKK causes degradation of IκBα and release of NF-κB for nuclear translocation. Again, Zfra is able to bind and sequester NF-κB in the cytoplasm. In **Step 5 and 6**, p53 is a downstream effector of TNF signaling [8,22-24]. TNF induces NF-κB activation, and then NF-κB activates p53 [22]. The non-ankyrin *C*-terminus of IκBα physically interacts with cytosolic p53 to prevent degradation *in vivo*, and the complex dissociates in response to TNF and apoptotic stress [25]. p53 is functionally associated with WOX1, and both proteins may induce apoptosis synergistically [3,4,6,8]. Thus, an *in vivo *complex of IκBα/p53/WOX1 is likely (Chang *et al*., submitted), and that Zfra may regulate the formation of this complex. We show that ectopic Zfra blocks UV light-induced p53 nuclear translocation or activation, suggesting a negative regulation for the cell death event. Ser46-phosphorylated p53 is known to play a critical role in apoptosis [8,25-29]. That is, Zfra may prevent cell death by blocking the apoptotic function of Ser46-phosphorylated p53. The IκBα/p53/WOX1 or p53/WOX1 may translocate to the mitochondria (Chang *et al*., submitted). Whether this event is blocked by Zfra is unknown.

Presence of two cysteine residues in the amino acid sequence of Zfra suggests the likely presence of a dimeric form in cells [10]. Also, whether Zfra covalently interacts with specific proteins remains to be established. As determined by GST-pull down analysis, both TNF and UV light are able to increase self-association of Zfra, supporting the presence of dimers in *vivo *[10]. Zfra is inducible under stress conditions [10]. We show that at low levels Zfra enhances cell growth, and yet overexpressed Zfra induces cell death. Ectopic Zfra also blocked adherence-independent growth of L929 fibroblasts, suggesting that it may act as a tumor suppressor to block cancer growth and progression. Zfra is most abundant in the spleen, whereas its role in regulating immune cell differentiation and functions is unknown. However, it is reasonable to suggest that Zfra-regulated TNF signaling is likely to play a role in the lymphocyte differentiation. We also determined that absent expression of Zfra is found in several breast and prostate cancer cell lines [10], implying that Zfra deficiency may provide a growth advantage for cancer cells. Majority of zinc finger proteins participate in gene transcription and embryonic development [30-32]. Synthetic peptides have been shown promises in therapy to modulate the transcriptional activities of endogenous zinc finger proteins [33,34]. Shortening and/or modification of the naturally occurring Zfra may be of therapeutic value in controlling cancer cell growth and death.

Zfra is distributed ubiquitously in cellular compartments. In response to TNF, a portion of cytosolic Zfra appears to translocate to the cell membrane area and binds TRADD. Death domain/death domain interaction is responsible for TRADD binding with p55-TNFR, FADD and RIP. Whether Zfra binds to the death domain is unknown. Sequence alignment analysis shows a common structural motif in TRADD, JNK1, NF-κB, and the *N*-terminal WW domain and *C*-terminal SDR domain of WOX1. Whether this motif interacts with Zfra remains to be established.

WWOX/WOX1 is known to interact with p53, p73, JNK1, AP-2γ, and ErbB4 [3,6,35-37], suggesting its role of the regulation of gene transcription. Both *in vivo *and *in vitro *studies have shown that overexpressed WWOX1/WOX1 suppresses tumor growth in nude mice, and induces apoptosis in cultured cells [3,6,8,9,38,39]. Overexpressed WW and SDR/C domains induce cell death [3]. Most interestingly, the *C*-terminal tail of WOX1 is needed to work together with SDR in causing cell death. In stark contrast, non-invasive breast and prostate cancers may have upregulated expression of WWOX/WOX1, Tyr33 phosphorylation and WWOXv2/WOX2 (and other family proteins if present) [40-42]. Downregulation of WOX1 and WOX2 promotes neurodegeneration in Alzheimer's disease, suggesting its protective role against neurodegeneration [43]. FOR (WWOX/WOX1) is shown to protect against the effects of ionizing radiation in *Drosophila *[44]. These observations support a pro-survival role of WOX1 *in vivo *[4].

In domain/domain mapping by yeast two-hybrid analysis, Zfra was shown to bind the *N*-terminal Tyr33-phosphorylated WW domain of WOX1. Alteration of this phosphorylation site abolishes the binding interaction. Also, it interacts with the *C*-terminal SDR domain of WOX1. Since Zfra is a small peptide, its simultaneous binding to these domains in WOX1 under physiologic and stress conditions is very likely. Our supporting evidence shows that stress stimuli induce phosphorylation of WOX1 at Tyr33 and Zfra at Ser8, and that the Zfra/WOX1 complex co-translocates to the mitochondria to regulate cell death via the mitochondrial pathway (Hsu *et al*., submitted). Also, in this study we found that Zfra enhances the apoptotic activity of SDR domain and the *C*-terminal tail of WOX1.

Overexpressed Zfra may nullify the apoptotic activities of WOX1 and p53. An apparent mechanism is that overexpressed Zfra sequesters transcriptional factors in the cytoplasm. For example, ectopic Zfra restricts nuclear localization of endogenous WOX1, p53, ERK, and NF-κB (p65), indicating that an upregulated level of Zfra may control the transcriptional system in cells under stress conditions.

Taken together, we have identified a molecular pathway underlying Zfra regulation of TNF cytotoxic function. Zfra interacts with TRADD and binds downstream NF-κB, p53, JNK1 and WOX1 of the TNF signal pathway, thereby either enhancing or restricting TNF-mediated cell death.

## Methods

### Cell lines, antibodies and chemicals

Majority of our experiments were performed using murine L929 fibroblasts, monkey kidney COS7 fibroblasts and human Molt4 T lymphocytes for ectopic gene expression. In addition, the following cell lines were cultured and tested for their sensitivity to Zfra-mediated death: 1) human neuroblastoma SK-N-SH cells, 2) human ovarian ME180 cells, 3) human breast MDA-MB-231 and MCF7 cells, 4) human embryonic kidney HEK-293 fibroblasts, 5) human prostate DU145 cells, and 6) mink lung epithelial Mv1Lu cells. Recombinant TNF-α was from PeproTech and R&D Systems. We have produced specific antibodies against the *NH*- and *COOH*-termini of WOX1, recombinant GST-WOX1 and Tyr33-phosphorylated WOX1 [3,6,43,45], synthetic Zfra peptide [10], and recombinant GST-Zfra [10]. The following specific antibodies were from Santa Cruz Biotechnology: p-JNK1 (phospho-JNK1 at Thr183/Tyr185), WWOX, NF-κB, IκBα, FADD, and p53. Anti-WWOX IgG, N-19 and P-20, were kind gifts of Santa Cruz Biotechnology. α-Tubulin antiserum was from Accurate Chemical, and anti-RIP from Pharmingen/BD Biosciences.

### Zfra and WOX1 cDNA constructs and transient gene expression

Zfra was constructed in CMV-based mammalian expression vectors pCR3.1 (Invitrogen) and pEGFPC1 (enhanced green fluorescent protein; cloning site EcoR1; Clontech) [10]. Murine full length WOX1, Y33R mutant, WW domains, SDR domain, and SDR domain with a *C*-terminal tail were constructed in pCR3.1, pEGFPC1 and pECFGC1 vectors [3,6,43]. Human p53 and p53(ΔS46) cDNAs were constructed in pDsRed-N1 (Clontech), as described [8]. Where indicated, cell lines were transfected with Zfra, WOX1 or p53 constructs, or empty vectors (15–600 ng/well) by calcium phosphate (CaPO4) or electroporation [3,6,8,10,43]. In electroporation, cells were transfected with the indicated expression constructs in the presence of albumin to enhance gene expression and reduce electric shock-induced accidental cell death [8]. Post transfection for 24–48 hr, the extent of cell growth or death was examined by crystal violet staining, cell cycle analysis by propidium iodide staining and fluorescence activated cell sorting (FACS), Annexin V assay, and DNA fragmentation analyses [3,6,8,43].

### Site-directed mutagenesis

The conserved phosphorylation site Ser8 in Zfra was mutated using QuikChange site-directed mutagenesis Kit (Stratagene). PCR primers for S8G-Zfra mutant were designed as follows: 1) S8G forward, GCAGAAGGTCGTCTGGCTGTAAATATTGTGA; 2) S8G reverse, TCACAATATTTACAGCCAGACGACCTTCTGC.

### Soft agarose colony survival assay

Adherence-independent or transforming cell growth was performed in a soft agarose colony survival assay [21]. Briefly, L929 cells were electroporated with expression constructs of EGFP-Zfra or EGFP alone. These cells were plated at a density of 3 × 10^4 ^cells/35-mm dish in triplicate in RPMI 1640, 10% fetal bovine serum, 0.8% agarose, and 10 mM Hepes. Dishes were incubated in a humidified CO2 incubator at 37°C for 3 weeks. The resulting colonies were stained with a soluble tetrazolium-based MTS cell proliferation reagent (Promega), and live colonies (with brown color) were counted [21].

### Electroporation, immunoprecipitation, and GST-Zfra pull-down assays

Cells were suspended in serum-free culture medium, containing 500 μg/ml bovine albumin, and electroporated with an EGFP-Zfra or EGFP construct (20 μg DNA/3 × 10^6 ^cells; 180 volt and 50 msec; BTX ECM 830 Square Wave Electroporator; Genetronics) [3,6,8,43]. Albumin enhanced both the transfection efficiency and gene expression by 3–5 folds. These cells were cultured 24–48 hr and then exposed to UV light or TNF-α for indicated times. Cytosolic and nuclear protein fractions were prepared using an NE-PER nuclear and cytosolic extraction kit (Pierce), followed by quantification, SDS-PAGE and Western blotting [3,6,8,43]. Where indicated, we performed 1) immunoprecipitation using specific antibodies against Zfra, and 2) pull-down assays using glutathione-Sepharose 4B beads coated with GST (Glutathione S-Transferase) or GST-Zfra to examine the presence of Zfra-binding proteins [3,6,8,10,43].

### Cytoplasmic yeast two-hybrid analysis

Ras rescue-based yeast two-hybrid analysis (CytoTrap, Stratagene) was performed as described [3,6,8]. Briefly, a cytosolic Sos-tagged bait protein is designed for binding to a cell membrane-anchored target protein (tagged with a myristoylation signal). This binding activates the Ras signal pathway in yeast and allows mutant yeast *cdc25H *to grow at 37°C using a selective agarose plate containing galactose. Without binding, yeast cells cannot grow at 37°C. We made the following bait constructs (in pSos vector): 1) a murine full-length WOX1, 2) the first WW domain and an NH2-terminal WW domain area of WOX1, 3) a COOH-terminal SDR domain area of WOX1 and a mitochondria-targeting region in this domain, and 4) a Y33R mutant in the WW domain area [3,6,8]. For target, a construct for Zfra was made in pMyr vector. Where indicated, full-length TRADD and RIP cDNAs were subcloned in pMyr (as target) or pSos (as bait). TRADD and RIP cDNAs were gifts of Dr. D. Goedell of Tularik/Amgen. Control constructs for the binding experiments were human MafB (in both pMyr and pSos) [3,6,8].

## Authors' contributions

QH carried out molecular analysis of protein/protein interactions. LJH performed protein-binding interactions and manuscript preparation. LS participated in preparing DNA expression constructs, cell growth and death assays, and necessary reagents for experiments. NP and JM participated in preparing constructs. NSC conceived of the study and experimental design, carried out key experiments, and wrote the manuscript. QH, LJH and NSC read and approved the final manuscript.
